# Associations of socioeconomic and religious factors with health: a population-based, comparison study between China and Korea using the 2010 East Asian social survey

**DOI:** 10.1186/s12889-018-6380-y

**Published:** 2019-01-08

**Authors:** Roeul Kim, Woojin Chung

**Affiliations:** 1 Labor Welfare Research Institute, Korea Workers’ Compensation and Welfare Service, Seoul, South Korea; 2 Department of Health Policy and Management, Graduate School of Public Health, Yonsei University, 50 Yonsei-ro, Seoul, Seodaemun-gu 120-752 South Korea; 3 Institute of Health Services Research, Yonsei University, Seoul, South Korea

**Keywords:** Socioeconomic factors, Religion, Self-rated health, Cross-national comparison, China, Korea

## Abstract

**Background:**

Cross-national comparisons of the associations of socioeconomic and religious factors with health can facilitate our understanding of differences in health determinants between countries and the development of policies to reduce health differentials appropriate to each country. However, very few such studies have been conducted in East Asia.

**Methods:**

This study set out to compare the associations of socioeconomic and religious factors with health in China and Korea using the 2010 East Asian Social Survey, which was based on nationally representative samples. The study participants included 4980 individuals, 3629 in China and 1351 in Korea, aged ≥20 years. The dependent variable, individuals’ self-rated health, was categorized into poor, good, and excellent. Socioeconomic (education, employment, household income, and self-assessed social class) and religious factors (affiliation) were used as independent variables of interest. A multinomial logistic regression was performed with and without adjustments for factors such as demographics, health-related risks, the health system, and social capital.

**Results:**

According to the results, China had a higher proportion of individuals who reported excellent health than did Korea (57.4% vs. 52.0%). After adjusting for all studied confounders, we found that the employment, household income, and social class gradient in health were significant in China, whereas the education and religion gradients in health were significant in Korea. For example, the odds ratio for poor health versus excellent health among those in the highest social class was 0.47 (95% CI, 0.27–0.84), compared to that of people in the lowest social class in China; and this odds ratio in people with college education or higher was 0.28 (95% CI, 0.14–0.59) compared to that of people with elementary school education or lower in Korea.

**Conclusions:**

These findings demonstrate the important role of socioeconomic and religious factors in health in China and Korea as well as clear differences in this regard. Further cross-national studies are needed to provide a better understanding of the relationship between socioeconomic and religious factors and health and to draft appropriate health improvement policies in both countries.

**Electronic supplementary material:**

The online version of this article (10.1186/s12889-018-6380-y) contains supplementary material, which is available to authorized users.

## Background

A large body of research has shown that socioeconomic factors have a significant impact on health within countries [1–3]. The pattern of health differentials within countries can be shaped by national health promotion policies, social security systems, delivery of healthcare services, and economic and political environments [4, 5]. Therefore, a comparative analysis of the association between socioeconomic factors and health in countries with different historical and economic backgrounds may provide useful evidence for addressing health differentials [5, 6].

Many previous studies have compared the association between socioeconomic factors and health among Western countries [5–10]. These studies have shown that such associations vary in strength across countries. Using data from the Collaborative Research on Ageing in Europe (COURAGE) survey conducted in Finland, Poland, and Spain, Freeman et al. [10] found a significant association between depression and socioeconomic status in all countries. Socioeconomic status was computed using the combined scores of the total number of years of education and the quintiles of the country-specific income level of the household. After adjusting for confounders, the odds of depression decreased significantly for every unit increase in the SES index for Finland, Poland, and Spain. Additionally, higher education significantly decreased the odds of depression in each country, but income did not [10].

However, very few studies have compared the association between socioeconomic factors and health in East Asian countries [11–14]. Among these, Hanibuchi et al. [11] explored the relationship between socioeconomic status and health in China, Japan, Korea, and Taiwan. However, their study had the notable limitation of relying on the 2006 East Asian Social Survey, which lacked information on disease status and health-related behaviors and therefore allowed only one control variable (age) in the analysis. Moreover, using cross-sectional surveys conducted in 2003, Nomura et al. [13] investigated the effects of socio-demographic factors on health in Korea and Taiwan. However, the sampling methods used in the two countries differed, thus preventing cross-country comparisons of the results. In addition, their research focused on the effects of social capital and demographic factors on health, rather than the effects of a broad range of socioeconomic factors.

Meanwhile, previous studies have demonstrated the protective effect of religion on the health status of individuals in Western countries [15–17]. Nicholson et al. [15] considered whether attendance at religious services was associated with better self-rated health in a range of European countries and reported an association between less frequent attendance of religious services and poor health in Europe, although they emphasized the importance of taking individual and contextual factors into account. However, the association between religion and health in Asian countries may be different from that in Western countries. For example, in China, religion is unpopular and church attendance is not common [18]. China has a population of about 1.3 billion, but only roughly 100 million of them (approximately 7.7%) are claimed to adhere to Buddhism, Taoism, Islam, Catholicism, or Protestant Christianity to at least some extent [19]. When the proportion of religious people is as low as in China, there are not enough religious people proportionately in the population to reflect the impact of religion on unhealthy behaviors [20]. Nevertheless, very few cross-national studies have examined the effect of religion on health in East Asian countries.

The aim of this study is to compare the associations of socioeconomic and religious factors with self-rated health in China and the Republic of Korea (hereafter, Korea). In our study, socioeconomic determinants were assessed based on education levels, employment, household income, and social class. Cross-national comparative research on health outcomes between China and Korea is of particular interest. China and Korea share many similarities in terms of Confucian culture, family structure, lifestyle, and geographical location [21], but the two countries also differ in important ways, namely, government structures, income distribution, social security systems, the delivery of healthcare services, social inequality, and religion [14]. Meanwhile, cross-national comparative research on health has not always been possible because of a lack of comparable data. To overcome these problems, we analyzed a dataset from the 2010 East Asian Social Survey (EASS), a cross-national survey consisting of nationally representative samples from China and Korea. To the best of our knowledge, no research to date has explicitly compared the associations between socioeconomic and religious factors and health in China and Korea.

## Methods

### Data source and study sample

The data used in this study were obtained from the 2010 EASS, which is an East Asian version of the European Social Survey. The EASS has been viewed as unique, a result of substantial efforts by East Asian countries to establish an internationally comparable database on social issues. The EASS was conducted on the basis of a vigorously controlled study protocol, including standard procedures for translating the measures into different languages and for collecting and controlling the data, thus enabling direct cross-national comparisons in a coordinated research setting. In the EASS, China and Korea shared a common module in a General Social Survey-type questionnaire, and each country conducted a survey of a nationally representative sample selected using a multistage stratified random sampling method.

In particular, the 2010 EASS included a health module concerning health status (self-rated health, chronic disease, etc.), health-related behaviors (smoking, drinking, exercise, etc.), caregiving and receiving care, health and social security insurance, social support and trust, epidemiology, family care need and care management, and so forth. The survey was administered face-to-face at respondents’ homes by trained interviewers from June to December, 2010. Valid response rates of 73.0 and 63.0% were obtained for China and Korea, respectively. Participants’ ages ranged from 18 to 98 years. The survey methodology has been described in an online report (http://www.eassda.org/).

Of the 5327 individuals aged ≥20 years, we excluded 347 (6.5%) due to missing values; however, we found no significant differences between the datasets before and after the exclusion (*p* < 0.827 for gender; *p* < 0.867 for age). Finally, we analyzed data from 4980 individuals, 3629 of whom were in China and 1351 in Korea.

The EASS data archive provides publicly available data from anonymous respondents. Verbal informed consent was obtained from all participants due to the limited time for survey interviews, and waivers of written consent were authorized by an ethics committee. Ethical approval for this study was granted by the institutional review board of the Graduate School of Public Health, Yonsei University, Seoul, Korea.

### Measures and variables

The dependent variable was individuals’ self-rated health. Respondents were prompted to answer the question, “How would you rate your health?” on a 5-point scale (excellent, very good, good, fair, and poor). The responses were grouped into the following three categories because of the sparseness of observations in each category: “poor” (“poor” or “fair”), “good” (“good”), and “excellent” (“very good” or “excellent”).

The independent variables of interest included socioeconomic factors (education, employment, household income, and self-assessed social class) and religious affiliation. Respondents’ education levels were classified into the following four categories: elementary school/lower (no formal qualification or elementary school), junior high school, senior high school, and college/higher (junior college, university, or graduate school completed). The highest education level reported by each individual was used as the indicator of education. Employment status was dichotomized into “employed” and “not employed” (having no current work income). Household income, which was a continuous variable, was divided by the square root of household size in order to adjust for household size [22], and categorized into income quartiles. To prevent any risk of bias, we included the 12.9% of respondents who did not report household income in the “missing income” category. Considering self-assessed social class, the following question was posed in the survey: “In our society, there are groups that tend to be toward the top and groups that tend to be toward the bottom. Below is a scale that runs from bottom to top. Where would you put yourself on this scale?” Available choices were on a 10-point scale from 1 (lowest) to 10 (highest). We converted the 10-point scale into a 5-point scale and merged the two highest categories because of the sparsity of cases in these groups. As a result, we obtained the following four categories of the variable of self-assessed social class: lowest, low, high, and highest. Individuals were also categorized on the basis of religious affiliation, namely, Christian, Buddhist, and others (Islam, Hinduism, atheism, agnosticism, and other religions). The Christian group included individuals who were affiliated with Roman Catholicism, Protestantism, Christian Orthodoxy, and other Christian religions.

As potential confounders, we considered various factors regarding (1) demographics, (2) health-related risks and the healthcare system, and (3) social capital. Demographic factors included gender, age, and marital status. Respondents were divided into six age groups, namely, 20–29, 30–39, 40–49, 50–59, 60–69, and ≥ 70 years. For the marital status variable, individuals were divided into three categories, married, never married, and formerly married (widowed, divorced, or separated). We removed unmarried individuals cohabiting with their partners because this category included only 52 individuals (0.5%).

Health-related risks and healthcare system factors include chronic disease, current smoking habits, drinking frequency, body mass index (BMI), physical exercise, health insurance, and unmet medical needs. The following variables were dichotomized: chronic disease (“having a chronic disease” or not), current smoking habits (“smoking a few times a year or more” or not), physical exercise (“doing physical exercise for at least 20 minutes a few times a year or more frequently” or not), health insurance (“having health insurance” or not), and unmet medical needs (“having healthcare needs in the past 12 months but did not receive care” or not). Drinking frequency was categorized into two groups, frequent (drinking daily or several times a week) and none or infrequent (drinking less than several times a month and not drinking). BMI was categorized as underweight (BMI < 18.5), normal weight (18.5 ≤ BMI < 25), overweight (25 ≤ BMI < 30), and obese (BMI > 30).

Social capital factors include generalized trust, emotional support, and instrumental support. Generalized trust was measured by the degree to which respondents agreed with the statement, “Generally, you can trust other people.” The response options were as follows: “highly trust,” “trust,” “do not trust,” and “do not trust at all.” This category was dichotomized, with the first two alternatives combined as “highly trust” and the latter two as indicating low trust. Emotional support was assessed with the question, “During the past 12 months, did people listen to your personal problems or concerns when you needed it?” The answers were grouped into three, namely, “yes,” “no,” and “do not have such needs.” Instrumental support was assessed with the question, “During the past 12 months, did people take care of your household chores (housework, childcare, and nursing care) when you needed it?” The answers were grouped into three, namely, “yes,” “no,” and “do not have such needs.”

### Analytic procedures

A three-step analysis was performed. First, the demographic factors of Chinese and Korean respondents were compared using *χ*^2^ tests. Second, differences in the proportion of individuals reporting excellent, good, or poor self-rated health were examined for each socioeconomic and religious factor for each country using *χ*^2^ tests. Third, multinomial logistic regression analysis, given the three categories of self-rated health, was used to assess the associations of socioeconomic and religious factors with self-rated health both without and with a full set of studied potential confounders within and between China and Korea.

While the ordinal nature of self-rated health would suggest using ordinal regression, multinomial logistic regression was appropriate in view of the violation of the proportional odds assumptions of ordinal regression. In this study, multinomial logistic regression was used to predict the risks of reporting either poor health or good health versus excellent health (reference). The adjusted odds ratios with 95% confidence intervals (OR, 95% CI) indicated the associations of the independent variables with good health versus excellent health (Table 3) or with poor health versus excellent health (Table 4).

The regression models were run for each country separately to explore possible national differences in the effects of socioeconomic and religious factors on health. Variance inflation factors (VIFs) for the independent variables were also within acceptable limits (VIF < 3.4), indicating no serious problems of collinearity. All data analyses were performed using SAS, version 9.2 (SAS Institute Inc., Cary, NC, USA).

## Results

Table 1 shows the distribution of each variable in China and Korea. China shows a higher proportion of individuals who reported excellent health than did Korea (57.4% versus 52.0%). The remaining factors for which China showed a higher proportion than Korea are as follows: elementary school education or lower; junior high school education; lowest social class; lower social class; other religions (Islam, Hinduism, atheism, agnosticism, and other religions); the ages of 40–49, 50–59, and 60–69 years; married; having a chronic disease; current smoker; being underweight; being obese; engaging in physical exercise; having no health insurance; having an unmet medical care need; having high generalized trust; having emotional support; and having instrumental support.

**Table 1:** Percentage distribution of variables in the study in China and Korea

Variable	China	Korea	*p*-value
*Self-rated health*			**
Poor	18.6	22.3	
Good	24.0	25.7	
Excellent	57.4	52.0	
*Education*			***
Elementary school/lower	37.2	13.0	
Junior high school	30.5	9.2	
Senior high school	17.1	30.1	
College/higher	15.2	47.7	
*Employment*			
Not employed^a^	34.9	37.3	
Employed	65.1	62.7	
*Household income*			
Lowest quartile	21.8	21.6	
2nd lowest quartile	21.3	21.9	
2nd highest quartile	22.1	22.5	
Highest quartile	21.9	23.3	
Missing	12.9	10.7	
*Self-assessed social class*			***
Lowest	19.5	10.8	
Low	34.7	31.4	
High	40.7	45.4	
Highest	5.1	12.4	
*Religion*			***
Christian	2.3	31.8	
Buddhist	4.3	24.3	
Others^b^	93.4	43.9	
*sex*			
Female	51.6	52.2	
Male	48.4	47.8	
*Age (years)*			***
20–29	12.9	16.3	
30–39	19.3	25.1	
40–49	24.8	24	
50–59	19.9	15.5	
60–69	13.4	10.6	
≥70	9.7	8.5	
*Marital status*			***
Never married	8.2	21.4	
Formerly married^c^	10.2	11.4	
Married	81.6	67.2	
*Chronic disease*			**
Yes	34.6	29.8	
No	65.4	70.2	
*Current smoking*			*
Yes	31.1	27.5	
No	68.9	72.5	
*Drinking frequency* ^*d*^			***
Frequent	17.3	30.4	
None or infrequent	82.7	69.6	
*Body mass index* ^*e*^			***
Underweight	11.0	6.8	
Nomal weight	67.7	70.2	
Overweight	18.8	20.8	
Obese	2.5	2.2	
*Physical exercise*			***
Yes	46.7	73.5	
No	53.3	26.5	
*Health insurance*			***
Yes	88.8	100.0	
No	11.2	0.0	
*Unmet medical need*			***
Yes	40.4	19.8	
No	59.6	80.2	
*Generalized trust*			***
Low	32.3	56.5	
High	67.7	43.5	
*Emotional support*			***
Yes	73.9	53.6	
No	11.2	12.9	
Do not have such needs	14.9	33.5	
*Instrumental support*			***
Yes	69.8	35.2	
No	11.7	29	
Do not have such needs	18.5	35.8	
N	3629	1351	

Note: †*p* < 0.10; *p < 0.05; **p < 0.01; ***p < 0.001

*P*-values are based on Chi-squared test statistic

^a^’Not employed’ includes the unemployed, the retired, the permanently disabled out of labour force, students and housewives

^b^’Formerly married’ includes widowed, divorced and separated

^c^’Frequent’ drinking includes daily or drinking several times a week. ‘None or infrequent’ drinking includes drinking less than several times a month and nondrinking

Table 2 reports on rates of self-rated health by each socioeconomic and religious factor in each country. The unadjusted association of each socioeconomic variable, excluding religion, with self-rated health was significant in both China and Korea. There is a steep education gradient in health. The data show that 31.4 and 59.7% of persons with elementary school education or lower in China and Korea reported poor health, compared to just 4.2 and 11.0% of their counterparts with college education or higher. Those who were not employed were more likely to report poor health in both countries. Moreover, those belonging to the lowest income quartile and those categorized as the lowest social class were much more likely to report poor health in both countries. Religion was associated with self-rated health, but only in Korea; Christians had better health than those whose self-reported religion was Buddhist or “others.” In addition, the rate of self-rated health varied between China and Korea in terms of socioeconomic factors, except for household income (*p* = 0.375).

**Table 2:** Percentage of poor, good, and excellent self-rated health by socioeconomic and religious factors in China and Korea

Variable	China				Korea			
	Poor	Good	Exc	*P*-value	Poor	Good	Exc	*P*-value
Education				***				***
Elementary school/lower	31.4	23.9	44.7		59.7	21.0	19.3	
Junior high school	13.6	25.2	61.2		30.6	33.9	35.5	
Senior high school	12.4	23.1	64.5		21.4	28.8	49.8	
College/higher	4.2	22.6	73.2		11.0	23.4	65.6	
Employment				***				***
Not employed^a^	26.2	29.2	44.6		32.5	25.8	41.7	
Employed	14.5	21.2	64.3		16.2	25.6	58.2	
Household income				***				***
Lowest quartile	33.0	23.5	43.5		43.8	26.7	29.5	
2nd lowest quartile	20.6	22.9	56.5		17.6	27.8	54.6	
2nd highest quartile	14.1	21.7	64.2		14.5	28.6	56.9	
Highest quartile	9.6	24.8	65.6		13.7	20.0	66.3	
Missing	13.7	29.1	57.2		23.5	25.5	51.0	
Self-assessed social class				***				***
Lowest	31.7	27.6	40.7		39.0	29.5	31.5	
Low	18.1	25.1	56.8		21.9	29.7	48.4	
High	13.0	22.0	65.0		18.9	23.5	57.6	
Highest	15.7	18.4	65.9		20.8	20.3	58.9	
Religion								***
Christian	24.7	24.7	50.6		20.7	20.5	58.8	
Buddhist	19.4	27.7	52.9		31.1	27.4	41.5	
Other	18.4	23.8	57.8		18.6	28.5	52.9	
N	3629				1351			

*Note*: *p < 0.05, **p < 0.01, ***p < 0.001

*P*-values are based on Chi-squared test statistic

^a^’Not employed’ includes the unemployed, the retired, the permanently disabled out of labour force, students and housewives

^b^’Others’ includes Islam, Hinduism, atheism, agnosticism, and other religions

Table 3 shows the odds ratios from the multinomial logistic regression predicting good health versus excellent health in both China and Korea. Table 4 shows the odds ratio for reporting poor health versus excellent health. To detect a change in the significance of socioeconomic and religious variables, the following two models were constructed: Model 1, with only socioeconomic and religious factors; and Model 2, with adjustments for the full set of studied potential confounders.

**Table 3:** Unadjusted and adjusted associations of socioeconomic and religious factors with good self-rated health in China and Korea (base outcome is excellent self-rated health): Results of multinomial logistic regression analyses

Variable	Model 1				Model 2			
	China		Korea		China		Korea	
	OR	(95% CI)	OR	(95% CI)	OR	(95% CI)	OR	(95% CI)
Education								
Elementary school/lower (ref.)	1.00		1.00		1.00		1.00	
Junior high school	0.82	(0.67–1.01)	1.00	(0.53–1.90)	1.05	(0.84–1.32)	0.77	(0.38–1.56)
Senior high school	0.70**	(0.55–0.90)	0.68	(0.39–1.17)	0.99	(0.75–1.32)	0.73	(0.38–1.40)
College/higher	0.67**	(0.51–0.88)	0.48**	(0.28–0.83)	1.22	(0.88–1.68)	0.62	(0.31–1.21)
Employment								
Not employed^a^ (ref.)	1.00		1.00		1.00		1.00	
Employed	0.51***	(0.43–0.60)	0.81	(0.60–1.08)	0.67***	(0.55–0.82)	0.75	(0.54–1.03)
Household income								
Lowest quartile (ref.)	1.00		1.00		1.00		1.00	
2nd lowest quartile	0.84	(0.65–1.09)	0.72	(0.46–1.10)	0.87	(0.66–1.14)	0.76	(0.48–1.21)
2nd highest quartile	0.77*	(0.59–1.00)	0.81	(0.52–1.27)	0.73*	(0.55–0.97)	0.86	(0.54–1.37)
Highest quartile	1.01	(0.76–1.32)	0.58*	(0.36–0.93)	0.95	(0.71–1.28)	0.59*	(0.35–0.98)
Missing	1.12	(0.84–1.50)	0.72	(0.43–1.22)	1.28	(0.94–1.75)	0.77	(0.44–1.33)
Self-assessed social class								
Lowest (ref.)	1.00		1.00		1.00		1.00	
Low	0.69**	(0.54–0.86)	0.84	(0.52–1.37)	0.70**	(0.55–0.90)	0.97	(0.57–1.63)
High	0.52***	(0.41–0.66)	0.61*	(0.38–0.99)	0.55***	(0.43–0.70)	0.75	(0.45–1.26)
Highest	0.42***	(0.27–0.65)	0.57	(0.31–1.04)	0.42***	(0.27–0.67)	0.66	(0.35–1.25)
Religion								
Christian (ref.)	1.00		1.00		1.00		1.00	
Buddhist	1.13	(0.59–2.17)	1.71**	(1.18–2.47)	1.34	(0.67–2.69)	1.72**	(1.16–2.55)
Others^b^	0.92	(0.54-1.59)	1.57**	(1.15–2.15)	1.07	(0.60–1.90)	1.66**	(1.19–2.32)
N	3629		1351		3629		1351	
Likelihood χ^2^ (degree of freedom)	560.55 (26)***		280.63 (26)***		1585.10 (70)***		614.46 (68)***	
−2 Log likelihood	6560.850		2485.06		5480.30		2151.24	

*Note: OR* odds ratio, *CI* confidence interval; *p < 0.05, **p < 0.01, ***p < 0.001

Model 2: adjusted for demographic, health-related risks/health care system, and social capital characteristics

^a^‘Not employed’ includes the unemployed, the retired, the permanently disabled out of labour force, students and housewives

^b^‘Others’ includes Islam, Hinduism, atheism, agnosticism, and other religions

**Table 4:** Unadjusted and adjusted associations of socioeconomic and religious factors with poor self-rated health in China and Korea (base outcome is excellent self-rated health): Results of multinomial logistic regression analyses

Variable	Model 1				Model 2			
	China		Korea		China		Korea	
	OR	(95% CI)	OR	(95% CI)	OR	(95% CI)	OR	(95% CI)
Education								
Elementary school/lower (ref.)	1.00		1.00		1.00		1.00	
Junior high school	0.38***	(0.30–0.47)	0.36**	(0.20–0.66)	0.64**	(0.48–0.85)	0.42*	(0.20–0.89)
Senior high school	0.36***	(0.27–0.48)	0.23***	(0.14–0.38)	0.72	(0.50–1.03)	0.49*	(0.25–0.96)
College/higher	0.15***	(0.09–0.23)	0.10***	(0.06–0.17)	0.49**	(0.28–0.84)	0.28***	(0.14–0.59)
Employment								
Not employed^a^ (ref.)	1.00		1.00		1.00		1.00	
Employed	0.37***	(0.31–0.45)	0.54***	(0.39–0.75)	0.58***	(0.45–0.75)	0.68	(0.45–1.01)
Household income								
Lowest quartile (ref.)	1.00		1.00		1.00		1.00	
2nd lowest quartile	0.65***	(0.50–0.84)	0.45***	(0.28–0.72)	0.70*	(0.51–0.96)	0.70	(0.40–1.23)
2nd highest quartile	0.48***	(0.36–0.64)	0.48**	(0.29–0.79)	0.47***	(0.33–0.66)	0.76	(0.42–1.38)
Highest quartile	0.47***	(0.34–0.66)	0.52*	(0.31–0.88)	0.49***	(0.33–0.73)	0.78	(0.42–1.46)
Missing	0.48***	(0.34–0.68)	0.54*	(0.31–0.94)	0.63*	(0.42–0.95)	0.72	(0.38–1.38)
Self-assessed social class								
Lowest (ref.)	1.00		1.00		1.00		1.00	
Low	0.50***	(0.39–0.64)	0.72	(0.43–1.21)	0.54***	(0.40–0.72)	1.11	(0.61–2.05)
High	0.37***	(0.28–0.47)	0.60*	(0.36–0.99)	0.42***	(0.31–0.58)	0.84	(0.46–1.53)
Highest	0.46**	(0.28–0.74)	0.78	(0.42–1.47)	0.47*	(0.27–0.84)	1.09	(0.52–2.28)
Religion								
Christian (ref.)	1.00		1.00		1.00		1.00	
Buddhist	0.96	(0.46–1.98)	1.72**	(1.16–2.55)	1.37	(0.58–3.23)	1.96**	(1.23–3.13)
Others^b^	0.89	(0.50-1.58)	1.08	(0.75–1.55)	1.26	(0.64–2.49)	1.34	(0.88–2.06)
N	3629		1351		3629		1351	
Likelihood χ^2^ (degree of freedom)	560.55 (26)***		280.63 (26)***		1585.10 (70)***		614.46 (68)***	
-2 Log likelihood	6560.850		2485.06		5480.30		2151.24	

*Note: OR* odds ratio, *CI* confidence interval, *p < 0.05, **p < 0.01, ***p < 0.001

Model 2: adjusted for demographic, health-related risks/health care system, and social capital characteristics

^a^‘Not employed’ includes the unemployed, the retired, the permanently disabled out of labour force, students and housewives

^b^‘Others’ includes Islam, Hinduism, atheism, agnosticism, and other religions

Additional file 1: Table S1 shows the odds ratios of good self-rated health by socioeconomic, demographic, risk/health care, and social capital factors within China and Korea (base outcome is excellent self-rated health). Additional file 1: Table S2 shows the odds ratio of poor self-rated health by socioeconomic, demographic, risk/health care, social capital factors within China and Korea (base outcome is excellent self-rated health).

Even after adjustment, a higher level of education significantly decreased the likelihood of reporting poor health versus excellent health in both countries. In particular, there was a steeper education gradient in health in Korea. The odds ratio for poor health was 0.28 (95% CI, 0.14–0.59) for Koreans with a college education or higher and 0.49 (95% CI, 0.25–0.96) for those with senior high school education, in comparison to their counterparts with elementary school education or lower (Model 2, Table 4). However, education level showed no significant association with good health versus excellent health in either China or Korea (Model 2, Table 3).

Only in China were employed persons much less likely than their unemployed counterparts to report either poor health or good health versus excellent health. For employed persons, the odds ratios for good and for poor health were 0.67 (95% CI, 0.55–0.82) and 0.58 (95% CI, 0.45–0.75), respectively (Model 2, Tables 3 and 4).

There was also a steep income gradient in health with respect to both 1) good health versus excellent health and 2) poor health versus excellent health in China, of which the latter was much clearer than the former. For Chinese subjects in the highest income quartile, the odds ratio for poor health was 0.49 (95% CI: 0.33–0.73) relative to their counterparts in the lowest income quartile. Meanwhile, in Korea this association between income and poor health was not significant after adjustments (Model 2, Table 4).

In the fully adjusted models, the association of social class with either 1) good health versus excellent health or 2) poor health versus excellent health was significant only in China, where a positive relationship was shown between social class and health. Compared to people in the lowest social class, the odds ratio for good health was 0.42 (95% CI: 0.27–0.67) and that for poor health was 0.47 (95% CI: 0.27–0.84) for people in the highest social class (Model 2, Tables 3 and 4).

The association between religion and health was significant only in Korea. For Buddhists, the odds ratio for good health was 1.72 (95% CI: 1.16–2.55), and 1.96 (95% CI: 1.23–3.13) for poor health (Model 2, Tables 3 and 4), compared to Christians. In addition, for Koreans in the “other” religion category, the odds ratio for good health was 1.66 (95% CI: 1.19–2.32) compared to Christians (Model 2, Table 3).

## Discussion

This study examined the association between socioeconomic and religious factors and self-rated health in China and Korea. We found that some socioeconomic and religious factors matter more in one country than the other. Employment, income, and social class seem to have generally stronger effects on health in China, while education and religion tend to have larger effects on health in Korea. Using multinomial logistic regression, we found that the findings are more pronounced with respect to poor health versus excellent health than for good health versus excellent health, as shown by the fact that the odds ratios reported in Table 4 tend to have greater statistical significance than do those in Table 3.

Our results reveal that in both China and Korea, education has a significant association with the odds of poor health versus excellent health, rather than with the odds of good health versus excellent health; these are consistent with the results of prior research indicating that education is strongly associated with health [23–25]. However, this education gradient in health was steeper in Korea. Considering that higher levels of education may confer knowledge and cognitive assets that are health protective [1], public policies to enhance education could provide the Korean people with greater health benefits.

In China, unlike Korea, employed persons were much less likely to report poor health than their unemployed counterparts, which is in line with the findings of earlier research [26–29]. The reason for this might be that employed persons were in the labor market because they were healthy [30]. However, this cannot explain the lack of evidence of an employment gradient in health in Korea. Another reason might pertain to differences in the accessibility of health determinants according to employment status in China. While Korea has implemented a universal public health insurance system providing all basic benefits that covers the whole population, China does not have a universal health insurance system, so that even insured people have different degrees of coverage depending on employment status and job characteristics [31, 32].

Meanwhile, we observed a steeper income gradient in health in China, suggesting the health impact of serious income inequalities in that country, which are currently viewed as a major problem in Chinese society. According to 2010 data, China’s Gini coefficient (0.481) places it among the countries with the highest levels of inequality in the world, not only higher than those of developed western countries but also higher than the Asian average (0.351) [33]. We may thus assume that although the healthcare system has been reformed, it is still difficult for people in low-income groups to access much-needed healthcare services [34]. People in the high-income bracket not only have a greater chance of accessing health care [35], but can also afford costly commercial insurance. In addition, income may also represent purchasing power or access to tangible resources (e.g., better housing, working conditions, food and health care, and increased social support and community cohesion) at both the individual and community level, with concomitant implications for health status [1]. Therefore, it seems that healthcare policy reform in China is necessary so as to reduce health inequalities resulting from income inequalities.

In the adjusted models, we found significant associations between social class and health in China but not in Korea. This finding is consistent with previous studies that have shown that self-rated health may be sensitive to social class differentials in some countries [11, 29, 36–38]. One possible explanation for this is a less equitable distribution of health-improving resources across social class in China than in Korea.

The association between religion and health was significant only in Korea, where Buddhists and people of other religions were more likely than Christians to report poor health. Many studies show that religion and spirituality are linked to health outcomes [15–17]. However, within this body of literature, it is unclear why people experience specific health benefits from religion [15, 39]. One possible explanation is that Christians disapprove more strongly of behavior that is potentially damaging to health [16]. However, we found that the association between religion and health was not significant in China. It is plausible that Chinese religions such as Buddhism and Taoism, as foundations of traditional Chinese culture, differ from Western religions in their conceptualizations of a supernatural being and afterlife, rituals, and organization, as well as health-influencing lifestyles [18]. In a study on religion, superstition, and Chinese suicide attempters, Zhang and Xu found that suicide attempters with high religiosity had a higher degree of suicide intent than did those with low religiosity or no religion, unlike the findings of Western studies [40]. They assumed that these findings might be due to Chinese religions and traditional cultural values. In Chinese religions there is no single god for people to worship, and these religions offer no social support system or coping mechanism as the majority of adherents do not meet regularly. Unlike the mainstream religions in the West, Chinese religions often seem to be associated with superstition, referred to as religious superstition (*zongjiao mixin*). To some Chinese people, being religious is equivalent to being superstitious, and death is seen as a solution to all problems and the beginning of a new life. Therefore, it is possible that those prone to extreme actions or in difficult circumstances are likely to think about starting a new life by ending their present miserable one quickly [40]. Meanwhile, our finding that religion is related to health in Korea and not in China may be due to a lack of statistical power. However, even in the additional analysis where we categorized religion into four groups (Christian, Buddhist, no religion, and others) and conducted multinomial logistic regression analyses, we found that the association between religion and health remained significant only in Korea (results can be provided on request).

To the best of our knowledge, the current study is the first to examine the associations of socioeconomic and religious factors with self-rated health in China and Korea using national data. The dataset permits direct cross-national comparisons through a coordinated research setting.

Our study had several clear limitations. First, objective health may be a better measure than self-rated health. Subjective reports of health status may be confounded by other variables, such as neuroticism or psychological distress, and may not correlate with the underlying pathology [41]. In addition, there is some uncertainty about what is being measured when using self-rated health as a health outcome [42]. However, measures of self-rated health have proven to be both reliable and valid health indicators across a wide range of age groups [43, 44] and predictors of objective health statuses [42–46], showing them to be closely associated with physicians’ diagnoses [47] and people’s future healthcare use use [48]. Second, it is important to bear in mind that the use of cross-sectional data precludes any definitive causal conclusions about the relationship between socioeconomic and religious factors with self-rated health. Third, the 2010 EASS had relatively low response rates. The response rates of 73.0% in China and 63.0% in Korea might have entailed non-response bias. We conducted an additional analysis comparing the distribution of individuals for each age category between the sample used in the present study and the sample of the national census for each country. According to these results, the difference in the proportion of individuals for each age category was less than 3 percentage points for each country, except for differences of 5.5 percentage points for individuals aged ≤29 in China and of 3.7 percentage points for individuals aged 30–39 in Korea (Additional file 1: Table S3). Fourth, there is a possibility that we could not completely control for health care system factors using only the two variables of health insurance and unmet medical need. With respect to health insurance, we only examined whether the participant had health insurance. Korea has a universal system of health care, a single insurer (the National Health Insurance Service), while China does not. Therefore, we could not control for the type of insurance or coverage depth (the scope and percentage of expenses reimbursed) in China. Fifth, health ratings may be influenced by cultural factors. For example, as stated in the 2016 OECD Health Data, Korea scored among the lowest of the member countries, as only 32.5% of the Korean population gave a good evaluation of their health in the self-rated health survey. In contrast, 88.1% of Americans rated their health as good, the highest rate among OECD members [49]. As has been noted elsewhere, when evaluating their health, respondents tend to draw upon different health aspects, including both physical and psychological well-being, and health behaviors [50]. However, previous research has shown that East Asians share similar patterns of perception and expression of, and response to, health [51]. Finally, we used only one variable pertaining to religion, religious affiliation. Because the EASS 2010 dataset does not provide additional information, we could not include other measures of religiosity such as the frequency of attendance at religious services, which has been used in previous studies [15, 16]. Further studies need to include such religious factors [15, 16].

## Conclusions

The present study found that socioeconomic and religious factors were associated with self-rated health in China and Korea, but that the patterns of the association differed between the two countries. The differences in health seem to be associated with employment, household income, and social class in China, whereas education and religion seem to be good predictors of health differentials in Korea. These inter-country differences may reflect systemic and cultural differences between China and Korea that impact social and economic inequalities, ultimately leading to differences in health. This study illuminates differences in health determinants between the countries of interest and may inform future health improvement in either country. Ultimately, there is a need for more sophisticated, cross-national studies to be conducted in more countries and to better facilitate those in China and Korea.

## Additional file

Additional file 1: **Tables S1**-**S2.** Odds ratios of good and poor self-rated health by socioeconomic, religious, demographic, risk/health care, and social capital factors within China and Korea (base outcome is excellent self-rated health); **Table S3.** Comparison of percentage distribution of individuals by age category between the study sample and the national census in China (2009) and Korea (2010). (DOCX 56 kb)

## Abbreviations

BMI: Body mass index
EASS: East Asian Social Survey
VIF: Variance inflation factor
