# Supplementary material for: Associations of socioeconomic and religious factors with health: a population-based, comparison study between China and Korea using the 2010 East Asian social survey
Source: BMC Public Health. 2019 Jan 8;19:35. doi: 10.1186/s12889-018-6380-y (PMC6323813; doi:10.1186/s12889-018-6380-y)
Supplement: Supplementary file 1 — Tables S1-S2. Odds ratios of good and poor self-rated health by socioeconomic, religious, demographic, risk/health care, and social capital factors within China and Korea (base outcome is excellent self-rated health); Table S3. Comparison of percentage distribution of individuals by age category between the study sample and the national census in China (2009) and Korea (2010). (DOCX 56 kb) [file 12889_2018_6380_MOESM1_ESM.docx]

**Additional file 1: Table S1.** Odds ratios of good self-rated health by socioeconomic, religious, demographic, risk/health care, and social capital factors within China and Korea (base outcome is excellent self-rated health)ᵃ

| Variable | Model 1 | | Model 2 | | Model 3 | | Model 4 | |
| --- | --- | --- | --- | --- | --- | --- | --- | --- |
|  | China | Korea | China | Korea | China | Korea | China | Korea |
| *Socioeconomic and religious* |  |  |  |  |  |  |  |  |
| **Education** |  |  |  |  |  |  |  |  |
| Elementary school/lower (ref.) | 1.00 | 1.00 | 1.00 | 1.00 | 1.00 | 1.00 | 1.00 | 1.00 |
| Junior high school | 0.82† | 1.00 | 1.07 | 0.86 | 1.07 | 0.80 | 1.05 | 0.77 |
| Senior high school | 0.70** | 0.68 | 0.92 | 0.69 | 1.01 | 0.73 | 0.99 | 0.73 |
| College/higher | 0.67** | 0.48** | 1.09 | 0.57† | 1.24 | 0.62 | 1.22 | 0.62 |
| **Household income** |  |  |  |  |  |  |  |  |
| Lowest quartile (ref.) | 1.00 | 1.00 | 1.00 | 1.00 | 1.00 | 1.00 | 1.00 | 1.00 |
| 2nd lowest quartile | 0.84 | 0.72 | 0.87 | 0.68† | 0.88 | 0.75 | 0.87 | 0.76 |
| 2nd highest quartile | 0.77* | 0.81 | 0.74* | 0.75 | 0.74* | 0.84 | 0.73* | 0.86 |
| Highest quartile | 1.01 | 0.58* | 0.94 | 0.50** | 0.97† | 0.57* | 0.95 | 0.59* |
| Missing | 1.12 | 0.72 | 1.14 | 0.72 | 1.30 | 0.76 | 1.28 | 0.77 |
| **Self-assessed social class** |  |  |  |  |  |  |  |  |
| Lowest (ref.) | 1.00 | 1.00 | 1.00 | 1.00 | 1.00 | 1.00 | 1.00 | 1.00 |
| Low | 0.69** | 0.84 | 0.67*** | 0.81 | 0.69** | 0.91 | 0.70** | 0.97 |
| High | 0.52*** | 0.61* | 0.51*** | 0.59* | 0.54*** | 0.70 | 0.55*** | 0.75 |
| Highest | 0.42*** | 0.57† | 0.39*** | 0.54* | 0.41*** | 0.61 | 0.42*** | 0.66 |
| **Employment** |  |  |  |  |  |  |  |  |
| Not employedᵇ (ref.) | 1.00 | 1.00 | 1.00 | 1.00 | 1.00 | 1.00 | 1.00 | 1.00 |
| Employed | 0.51*** | 0.81 | 0.67*** | 0.78 | 0.66*** | 0.75† | 0.67*** | 0.75† |
| **Religion** |  |  |  |  |  |  |  |  |
| Christian (ref.) | 1.00 | 1.00 | 1.00 | 1.00 | 1.00 | 1.00 | 1.00 | 1.00 |
| Buddhist | 1.13 | 1.71** | 1.17 | 1.66** | 1.32 | 1.75** | 1.34 | 1.72** |
| Other | 0.92 | 1.57** | 1.00 | 1.63** | 1.06 | 1.68** | 1.07 | 1.66** |
| *Demographic* |  |  |  |  |  |  |  |  |
| **Gender** |  |  |  |  |  |  |  |  |
| Female (ref.) |  |  | 1.00 | 1.00 | 1.00 | 1.00 | 1.00 | 1.00 |
| Male |  |  | 0.81* | 0.82 | 0.86 | 0.75 | 0.85 | 0.77 |
| **Age (years)** |  |  |  |  |  |  |  |  |
| 20-29 (ref.) |  |  | 1.00 | 1.00 | 1.00 | 1.00 | 1.00 | 1.00 |
| 30-39 |  |  | 1.75** | 1.75* | 1.64* | 1.71† | 1.64* | 1.72* |
| 40-49 |  |  | 2.31*** | 1.76† | 1.98*** | 1.69† | 1.99*** | 1.79† |
| 50-59 |  |  | 3.53*** | 2.75** | 2.51*** | 2.59** | 2.53*** | 2.69** |
| 60-69 |  |  | 4.22*** | 1.50 | 2.45*** | 1.40 | 2.37*** | 1.47 |
| ≥70 |  |  | 4.35*** | 1.39 | 2.31*** | 1.19 | 2.38*** | 1.19 |
| **Marital status** |  |  |  |  |  |  |  |  |
| Never married (ref.) |  |  | 1.00 | 1.00 | 1.00 | 1.00 | 1.00 | 1.00 |
| Married but singleᶜ |  |  | 0.97 | 0.84 | 0.92 | 0.74 | 0.96 | 0.74 |
| Married |  |  | 0.94 | 0.94 | 0.96 | 0.94 | 0.94 | 0.95 |
| *Risk/Health Care* |  |  |  |  |  |  |  |  |
| **Chronic disease** |  |  |  |  |  |  |  |  |
| Yes |  |  |  |  | 4.36*** | 2.39*** | 4.36*** | 2.34*** |
| No (ref.) |  |  |  |  | 1.00 | 1.00 | 1.00 | 1.00 |
| **Current smoking** |  |  |  |  |  |  |  |  |
| Yes |  |  |  |  | 1.02 | 1.71** | 1.01 | 1.73** |
| No (ref.) |  |  |  |  | 1.00 | 1.00 | 1.00 | 1.00 |
| **Current drinkingᵈ** |  |  |  |  |  |  |  |  |
| Heavy |  |  |  |  | 1.01 | 0.80 | 1.00 | 0.80 |
| None or moderate (ref.) |  |  |  |  | 1.00 | 1.00 | 1.00 | 1.00 |
| **BMI** |  |  |  |  |  |  |  |  |
| Underweight |  |  |  |  | 1.11 | 1.68† | 1.12 | 1.68† |
| Nomal weight (ref.) |  |  |  |  | 1.00 | 1.00 | 1.00 | 1.00 |
| Overweight |  |  |  |  | 0.85 | 1.27 | 0.84 | 1.27 |
| Obese |  |  |  |  | 1.24 | 3.35* | 1.25 | 3.25* |
| **Physical exercise** |  |  |  |  |  |  |  |  |
| Yes |  |  |  |  | 0.82* | 0.58** | 0.82* | 0.57** |
| No (ref.) |  |  |  |  | 1.00 | 1.00 | 1.00 | 1.00 |
| **Unmet medical need** |  |  |  |  |  |  |  |  |
| Yes |  |  |  |  | 1.11 | 2.02*** | 1.10 | 1.96*** |
| No (ref.) |  |  |  |  | 1.00 | 1.00 | 1.00 | 1.00 |
| **Health insurance** |  |  |  |  |  |  |  |  |
| Yes |  |  |  |  | 1.10 |  | 1.12 |  |
| No (ref.) |  |  |  |  | 1.00 |  | 1.00 |  |
| *Social Capital* |  |  |  |  |  |  |  |  |
| **Generalized trust** |  |  |  |  |  |  |  |  |
| low (ref.) |  |  |  |  |  |  | 1.00 | 1.00 |
| high |  |  |  |  |  |  | 0.79* | 0.81 |
| **Emotional support** |  |  |  |  |  |  |  |  |
| Yes (ref.) |  |  |  |  |  |  | 1.00 | 1.00 |
| No |  |  |  |  |  |  | 0.97 | 1.23 |
| Do not have such needs |  |  |  |  |  |  | 1.13 | 0.71 |
| **Instrumental support** |  |  |  |  |  |  |  |  |
| Yes (ref.) |  |  |  |  |  |  | 1.00 | 1.00 |
| No |  |  |  |  |  |  | 1.07 | 0.88 |
| Do not have such needs |  |  |  |  |  |  | 1.00 | 1.14 |
| Note. †p < 0.10; *p < 0.05; **p < 0.01; ***p < 0.001 ᵃColumns labelled C-K show statistically significant differences between China and Korea for each category of the variable.  ᵇ'Not employed' includes the unemployed, the retired, the permanently disabled out of labour force, students and housewives. ᶜ'Formerly married' includes widowed, divorced and separated. ᵈ'Heavy' drinking includes daily or drinking several times a week.  'None or Moderate' drinking includes drinking less than several times a month and nondrinking. | | | | | | | | |

**Table S2**. Odds ratios of poor self-rated health by socioeconomic, religious, demographic, risk/health care, social capital factors within China and Korea (base outcome is excellent self-rated health)ᵃ

| Variable | Model 1 | | Model 2 | | Model 3 | | Model 4 | |
| --- | --- | --- | --- | --- | --- | --- | --- | --- |
|  | China | Korea | China | Korea | China | Korea | China | Korea |
| *Socioeconomic and religious* |  |  |  |  |  |  |  |  |
| **Education** |  |  |  |  |  |  |  |  |
| Elementary school/lower (ref.) | 1.00 | 1.00 | 1.00 | 1.00 | 1.00 | 1.00 | 1.00 | 1.00 |
| Junior high school | 0.38*** | 0.36** | 0.59*** | 0.52* | 0.64** | 0.42* | 0.64** | 0.42* |
| Senior high school | 0.36*** | 0.23*** | 0.55*** | 0.47* | 0.70† | 0.49* | 0.72† | 0.49* |
| College/higher | 0.15*** | 0.10*** | 0.32*** | 0.27*** | 0.47** | 0.29*** | 0.49** | 0.28*** |
| **Household income** |  |  |  |  |  |  |  |  |
| Lowest quartile (ref.) | 1.00 | 1.00 | 1.00 | 1.00 | 1.00 | 1.00 | 1.00 | 1.00 |
| 2nd lowest quartile | 0.65*** | 0.45*** | 0.68** | 0.57* | 0.69* | 0.69 | 0.70* | 0.70 |
| 2nd highest quartile | 0.48*** | 0.48** | 0.45*** | 0.58* | 0.46*** | 0.73 | 0.47*** | 0.76 |
| Highest quartile | 0.47*** | 0.52* | 0.42*** | 0.55* | 0.47*** | 0.76 | 0.50*** | 0.78 |
| Missing | 0.48*** | 0.54* | 0.47*** | 0.60† | 0.62* | 0.69 | 0.63* | 0.72 |
| **Self-assessed social class** |  |  |  |  |  |  |  |  |
| Lowest (ref.) | 1.00 | 1.00 | 1.00 | 1.00 | 1.00 | 1.00 | 1.00 | 1.00 |
| Low | 0.50*** | 0.72 | 0.50*** | 0.83 | 0.53*** | 1.06 | 0.54*** | 0.11 |
| High | 0.37*** | 0.60* | 0.36*** | 0.64† | 0.41*** | 0.82 | 0.42*** | 0.84 |
| Highest | 0.46*** | 0.78 | 0.41*** | 0.85 | 0.47** | 1.04 | 0.47* | 1.09 |
| **Employment** |  |  |  |  |  |  |  |  |
| Not employedᵇ (ref.) | 1.00 | 1.00 | 1.00 | 1.00 | 1.00 | 1.00 | 1.00 | 1.00 |
| Employed | 0.37*** | 0.54*** | 0.58*** | 0.66* | 0.57*** | 0.65* | 0.58*** | 0.68† |
| **Religion** |  |  |  |  |  |  |  |  |
| Christian (ref.) | 1.00 | 1.00 | 1.00 | 1.00 | 1.00 | 1.00 | 1.00 | 1.00 |
| Buddhist | 0.96 | 1.72** | 1.05 | 1.77** | 1.30 | 1.97** | 1.37 | 1.96** |
| Other | 0.89 | 1.08 | 1.11 | 1.23 | 1.23 | 1.34 | 1.26 | 1.34 |
| *Demographic* |  |  |  |  |  |  |  |  |
| **Gender** |  |  |  |  |  |  |  |  |
| Female (ref.) |  |  | 1.00 | 1.00 | 1.00 | 1.00 | 1.00 | 1.00 |
| Male |  |  | 0.66*** | 0.71† | 0.96 | 0.82 | 0.95 | 0.90 |
| **Age (years)** |  |  |  |  |  |  |  |  |
| 20-29 (ref.) |  |  | 1.00 | 1.00 | 1.00 | 1.00 | 1.00 | 1.00 |
| 30-39 |  |  | 2.56** | 1.84† | 1.95† | 1.78 | 1.93† | 1.93† |
| 40-49 |  |  | 6.52*** | 2.55* | 4.09*** | 1.72 | 4.15*** | 2.17† |
| 50-59 |  |  | 10.55*** | 4.28*** | 4.67*** | 2.39† | 4.92*** | 2.83* |
| 60-69 |  |  | 13.12*** | 4.79*** | 4.21*** | 2.21 | 4.39*** | 3.08* |
| ≥70 |  |  | 16.57*** | 9.48*** | 4.40*** | 4.23* | 4.76*** | 5.50** |
| **Marital status** |  |  |  |  |  |  |  |  |
| Never married (ref.) |  |  | 1.00 | 1.00 | 1.00 | 1.00 | 1.00 | 1.00 |
| Married but singleᶜ |  |  | 0.72 | 0.85 | 0.69 | 0.71 | 0.70 | 0.73 |
| Married |  |  | 0.63 | 0.68 | 0.63 | 0.75 | 0.66 | 0.76 |
| *Risk/Health Care* |  |  |  |  |  |  |  |  |
| **Chronic disease** |  |  |  |  |  |  |  |  |
| Yes |  |  |  |  | 19.80*** | 10.09*** | 19.72*** | 9.69*** |
| No (ref.) |  |  |  |  | 1.00 | 1.00 | 1.00 | 1.00 |
| **Current smoking** |  |  |  |  |  |  |  |  |
| Yes |  |  |  |  | 0.95 | 1.55† | 0.94 | 1.51† |
| No (ref.) |  |  |  |  | 1.00 | 1.00 | 1.00 | 1.00 |
| **Current drinkingᵈ** |  |  |  |  |  |  |  |  |
| Heavy |  |  |  |  | 0.62** | 0.67 | 0.61** | 0.66† |
| None or moderate (ref.) |  |  |  |  | 1.00 | 1.00 | 1.00 | 1.00 |
| **BMI** |  |  |  |  |  |  |  |  |
| Underweight |  |  |  |  | 1.69** | 1.28 | 1.72*** | 1.26 |
| Nomal weight (ref.) |  |  |  |  | 1.00 | 1.00 | 1.00 | 1.00 |
| Overweight |  |  |  |  | 0.84 | 1.56* | 0.83 | 1.48† |
| Obese |  |  |  |  | 0.75 | 4.47* | 0.71 | 4.32* |
| **Physical exercise** |  |  |  |  |  |  |  |  |
| Yes |  |  |  |  | 0.52*** | 0.49*** | 0.52*** | 0.50** |
| No (ref.) |  |  |  |  | 1.00 | 1.00 | 1.00 | 1.00 |
| **Unmet medical need** |  |  |  |  |  |  |  |  |
| Yes |  |  |  |  | 1.16 | 3.60*** | 1.13 | 3.58*** |
| No (ref.) |  |  |  |  | 1.00 | 1.00 | 1.00 | 1.00 |
| **Health insurance** |  |  |  |  |  |  |  |  |
| Yes |  |  |  |  | 1.39† |  | 1.43† |  |
| No (ref.) |  |  |  |  | 1.00 |  | 1.00 |  |
| *Social Capital* |  |  |  |  |  |  |  |  |
| **Generalized trust** |  |  |  |  |  |  |  |  |
| low (ref.) |  |  |  |  |  |  | 1.00 | 1.00 |
| high |  |  |  |  |  |  | 0.77* | 0.69* |
| **Emotional support** |  |  |  |  |  |  |  |  |
| Yes (ref.) |  |  |  |  |  |  | 1.00 | 1.00 |
| No |  |  |  |  |  |  | 1.14 | 0.83 |
| Do not have such needs |  |  |  |  |  |  | 1.01 | 0.51† |
| **Instrumental support** |  |  |  |  |  |  |  |  |
| Yes (ref.) |  |  |  |  |  |  | 1.00 | 1.00 |
| No |  |  |  |  |  |  | 1.07 | 0.59* |
| Do not have such needs |  |  |  |  |  |  | 0.73 | 1.06 |
| Note. †p < 0.10; *p < 0.05; **p < 0.01; ***p < 0.001 ᵃColumns labelled C-K show statistically significant differences between China and Korea for each category of the variable.  ᵇ'Not employed' includes the unemployed, the retired, the permanently disabled out of labour force, students and housewives. ᶜ'Formerly married' includes widowed, divorced and separated. ᵈ'Heavy' drinking includes daily or drinking several times a week.  'None or Moderate' drinking includes drinking less than several times a month and nondrinking. | | | | | | | | |

| **Table S3.** Comparison of percentage distribution of individuals by age category between the study sample and the national census in China (2009) and Korea (2010) | | | | | | | |
| --- | --- | --- | --- | --- | --- | --- | --- |
| Age (years) | China | | |  | Korea | | |
|  | Study sample | Census | Difference |  | Study sample | Census | Difference |
| 20-29 | 12.9 | 18.4 | -5.5 |  | 16.3 | 17.6 | -1.3 |
| 30-39 | 19.3 | 20.9 | -1.6 |  | 25.2 | 21.5 | 3.7 |
| 40-49 | 24.8 | 23.1 | 1.7 |  | 24 | 22.7 | 1.3 |
| 50-59 | 19.9 | 18.5 | 1.4 |  | 15.5 | 18.2 | -2.7 |
| 60-69 | 13.5 | 10.8 | 2.7 |  | 10.6 | 10.8 | -0.2 |
| ≥70 | 9.7 | 8.3 | 1.4 |  | 8.5 | 9.2 | -0.7 |
| ***N*** | 3,629 | 1,010,894,500 |  |  | 1,351 | 38,931,267 |  |
| China: http://www.stats.gov.cn/english/statisticaldata/censusdata Korea: http://kosis.kr/eng | | | | | | | |
